# Neural correlates of distraction and conflict resolution for nonverbal auditory events

**DOI:** 10.1038/s41598-017-00811-7

**Published:** 2017-05-09

**Authors:** Hannah J. Stewart, Sygal Amitay, Claude Alain

**Affiliations:** 1 Rotman Research Institute, Baycrest Hospital & Department of Psychology, University of Toronto, Toronto, Canada; 2 Medical Research Council Institute of Hearing Research, The University of Nottingham, University Park, Nottingham, NG7 2RD UK; 3 School of Psychology, The University of Nottingham, University Park, Nottingham, NG7 2RD UK; 4 Communication Sciences Research Center, Cincinnati Children’s Hospital Medical Center, Cincinnati, OH 45229 USA; 5 Nottingham University Hospitals NHS Trust, Nottingham, UK

## Abstract

In everyday situations auditory selective attention requires listeners to suppress task-irrelevant stimuli and to resolve conflicting information in order to make appropriate goal-directed decisions. Traditionally, these two processes (i.e. distractor suppression and conflict resolution) have been studied separately. In the present study we measured neuroelectric activity while participants performed a new paradigm in which both processes are quantified. In separate block of trials, participants indicate whether two sequential tones share the same pitch or location depending on the block’s instruction. For the *distraction* measure, a positive component peaking at ~250 ms was found – a distraction positivity. Brain electrical source analysis of this component suggests different generators when listeners attended to frequency and location, with the distraction by location more posterior than the distraction by frequency, providing support for the dual-pathway theory. For the *conflict resolution* measure, a negative frontocentral component (270–450 ms) was found, which showed similarities with that of prior studies on auditory and visual conflict resolution tasks. The timing and distribution are consistent with two distinct neural processes with suppression of task-irrelevant information occurring before conflict resolution. This new paradigm may prove useful in clinical populations to assess impairments in filtering out task-irrelevant information and/or resolving conflicting information.

## Introduction

It is well accepted that attention is not a unitary phenomenon, but rather involves processes that may vary as a function of the task at hand^1, 2^. In auditory attention, internal goal-directed actions enable listeners to prioritize and select task-relevant sounds for further processing^3, 4^. In addition to enhancing processing of task-relevant stimuli, selective attention may also involve top-down suppression of task-irrelevant information to stay on task^5–8^, which would prevent information overload. Auditory attention also often requires processing incongruency in relation to the task demand (i.e. conflict resolution). For instance, prior behavioural studies have shown that listeners take longer to respond to the word ‘high’ when presented at a low pitch^9^. In most everyday listening situations, these two processes may be required to occur concurrently for successful goal-directed action. Yet prior research has typically evaluated these two skills separately. For instance, neuroimaging studies using dichotic listening paradigm focuses primarily on early selection mechanisms^10^ while those using an auditory analogue of the Stroop task focus on late selection process and conflict resolution^11, 12^. In the present study, we use electroencephalography (EEG) to investigate the neural correlates supporting suppression of distracting irrelevant information and conflict resolution using a new auditory paradigm, the Test of Attention in Listening (TAiL)^13^.

Within TAiL the listener has to decide if the trial’s two sequential tones were same or different regarding their features of frequency (attend-frequency task) *or* location (attend-location task). Through different combinations of the task-relevant and -irrelevant stimulus features (frequency and location) changing, and/or staying constant, the listener’s auditory *distraction* by the task-irrelevant stimuli feature can be calculated, along with the listener’s ability to deal with sensory *conflict* (i.e. when one of the stimuli features changes compared to both staying the same or changing)^13^.

Prior research using scalp-recording of visual event-related potentials (ERPs) have revealed a distracter positivity (Pd) modulation. This ERP modulation (i.e. component) has been found using mainly visual search paradigms where the target differs from the distracters in one feature (e.g. shape^14^; orientation^15^; colour^16^). It is thought to reflect the suppression of, and possibly the orientation to, irrelevant but potentially distracting lateralized stimulus^14–16^. The Pd has an onset ranging from 250–300 ms post-stimulus with a positive parietal placement, contralateral to the presentation of the distracter^14^. The Pd appears to be similar to another ERP modulation, the PTc (i.e. a positive modulation over temporal scalp region contralateral to the distracter), which is also thought to index the suppression of distracter stimulus. The PTc peaks at about 290–370 ms after stimulus onset and is largest over the temporal scalp region contralateral to the lateralized distracter^17^. The small differences in latency and amplitude distribution between these two ERP modulations could be attributed to the salience of the distracter stimulus^15, 18^. Further to these components, the rejection positivity (RP) has been used to describe a component in auditory-based studies akin to the Pd and PTc – with a frontocentral distribution occurring at 200–250 ms post-stimulus^6, 7, 19^.

A popular method to assess conflict resolution has been the Stroop paradigm. Prior visual Stroop studies have revealed a frontocentral negative difference wave component, the N450^20–23^. This component typically has an onset of 350 to 400 ms continuing to 500 ms with an origin in the medial dorsal area, specifically the anterior cingulate cortex^20^. Comparatively, very few EEG studies have explored auditory Stroop tasks, at this time we are only aware of two such studies^11, 12^. Both of these studies used verbal stimuli (e.g. “High” and “Low” or “Left” and “Right”) and found difference wave components similar in topography to visual Stroop tasks (frontocentral negativity). However, the ERPs presented earlier with an onset of 200 to 250 ms lasting until 500 ms and peaking at about 300 ms.

A later posterior sustained positivity (SP) was also identified at 500–800 ms for visual and auditory Stroop tasks^11, 20–23^. It has been proposed that this component, SP, reflects additional supramodal processing, such as word meaning^20^, or the control required to accurately respond to the trial^24^. Therefore we expect to find this component coinciding with the *conflict resolution* measure.

Whilst the behavioural outcomes of TAiL have consistently been shown across independent populations^13, 25^, the underlying neural underpinnings have not yet been investigated. Understanding TAiL’s neural correlates may help break down where in auditory processing task instruction modulates performance. As currently, behavioural results suggest that by simply changing the TAiL task instructions auditory attention processes change, despite the stimuli remaining constant^25^.

TAiL has been compared to other tests of attention using auditory and visual stimuli, namely the visual and auditory Attention Network Tests and the Test of Everyday Attention. Results indicate that TAiL’s attend-location task provides supramodal measures of selective attention, whilst the attend-frequency task provides auditory-specific measures^25^. This follows the dual-pathway theory of processing spatial and non-spatial stimuli features in the dorsal and ventral streams, respectively (see review: refs 26, 27). Within this theory it is suggested that non-spatial features are processed in a modality-specific fashion, and spatial features in a supramodal fashion^28–30^.

## Results

### Behavioural results

Repeated 2 × 2 ANOVAs (frequency: same, different; location: same, different) for each TAiL task showed significant effects for the measures of *distraction* and *conflict resolution*. For comparison with prior research, conflict resolution and distraction measures were examined for attend frequency and attend location, separately.

#### Conflict resolution

**Figure 1:**
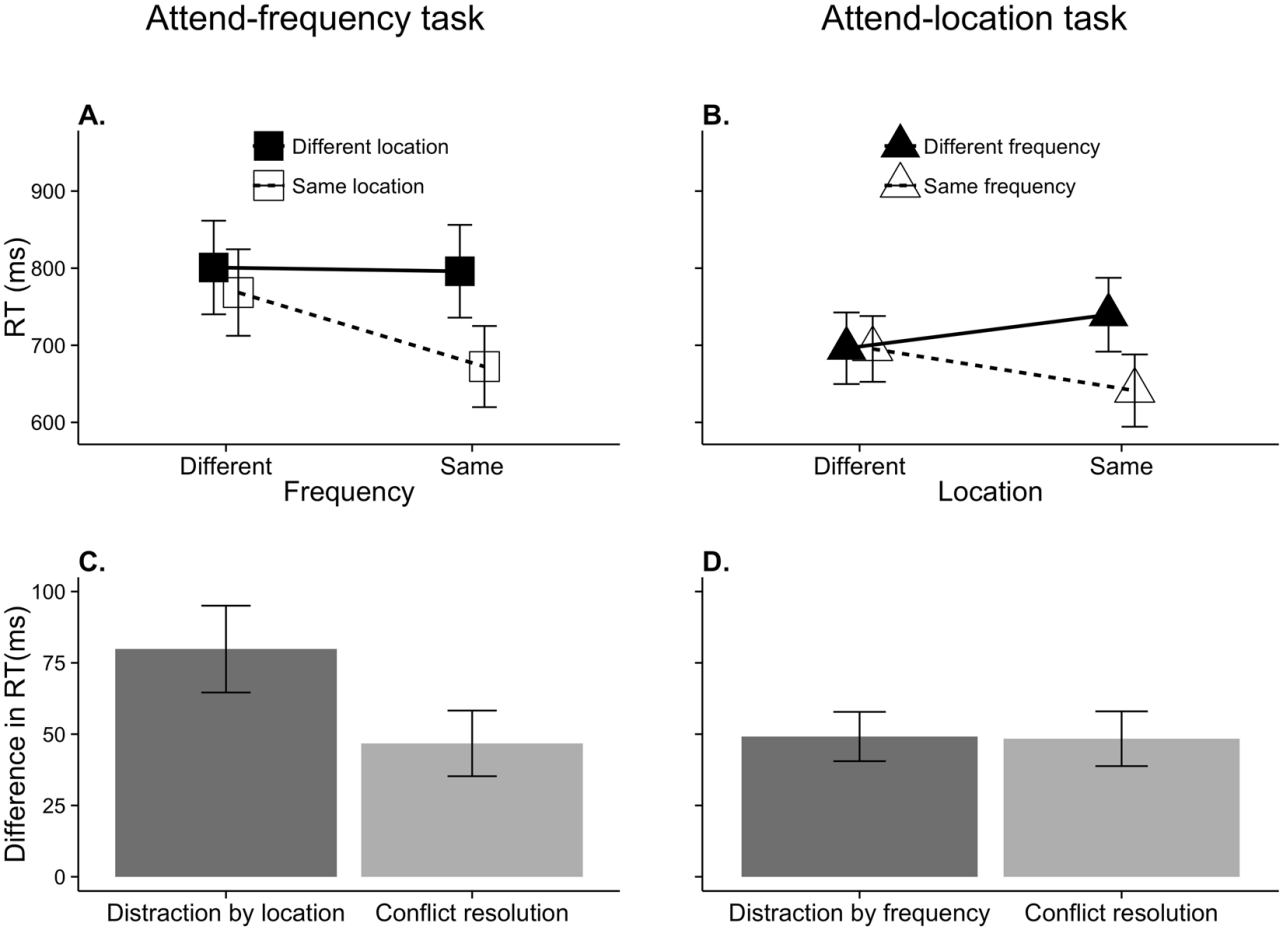
Group mean response times for the (**A**) attend-frequency and (**B**) attend-location TAiL tasks. Group mean measures of distraction and conflict resolution for the (**C**) attend-frequency and (**D**) attend-location TAiL tasks. The error bars represent the standard error of the mean.

In both tasks (Fig. 1A,B; Table 1), participants were faster when both stimulus features stayed the same or changed than when only one of the stimulus features changed (frequency × location interaction: attend-frequency task: *F*(1, 15) = 17.62, *p* < 0.001, ${{\rm{\eta }}}_{{\rm{p}}}^{2}$ = 0.54; attend location task: *F*(1, 15) = 28.16, *p* < 0.001, ${{\rm{\eta }}}_{{\rm{p}}}^{2}$ = 0.65). Post-hoc analysis of this interaction shows slower response times (RTs) when the task-irrelevant feature changed than when it stayed constant in trials where the task-relevant feature also stayed constant (attend-frequency: *t*(15) = −7.23, *p* < 0.001; attend-location: *t*(15) = −7.43, *p* < 0.001), but not when the task-relevant feature changed (attend-frequency: *t*(15) = −1.68, *p* = 0.11; attend-location: *t*(15) = −0.08, *p* = 0.94).

**Table 1:** Group mean and standard error of the mean response times from both TAiL tasks for different and same irrelevant feature changes, and incongruent and congruent feature changes.

	Attend-frequency task RT (ms)		Attend-location task RT (ms)	
	Mean	SEM	Mean	SEM
Different irrelevant feature	798.46	42.05	717.90	33.05
Same irrelevant feature	720.40	38.87	668.26	31.57
Incongruent	736.64	41.28	668.69	32.84
Congruent	782.22	40.55	717.46	31.61

**Figure 2:**
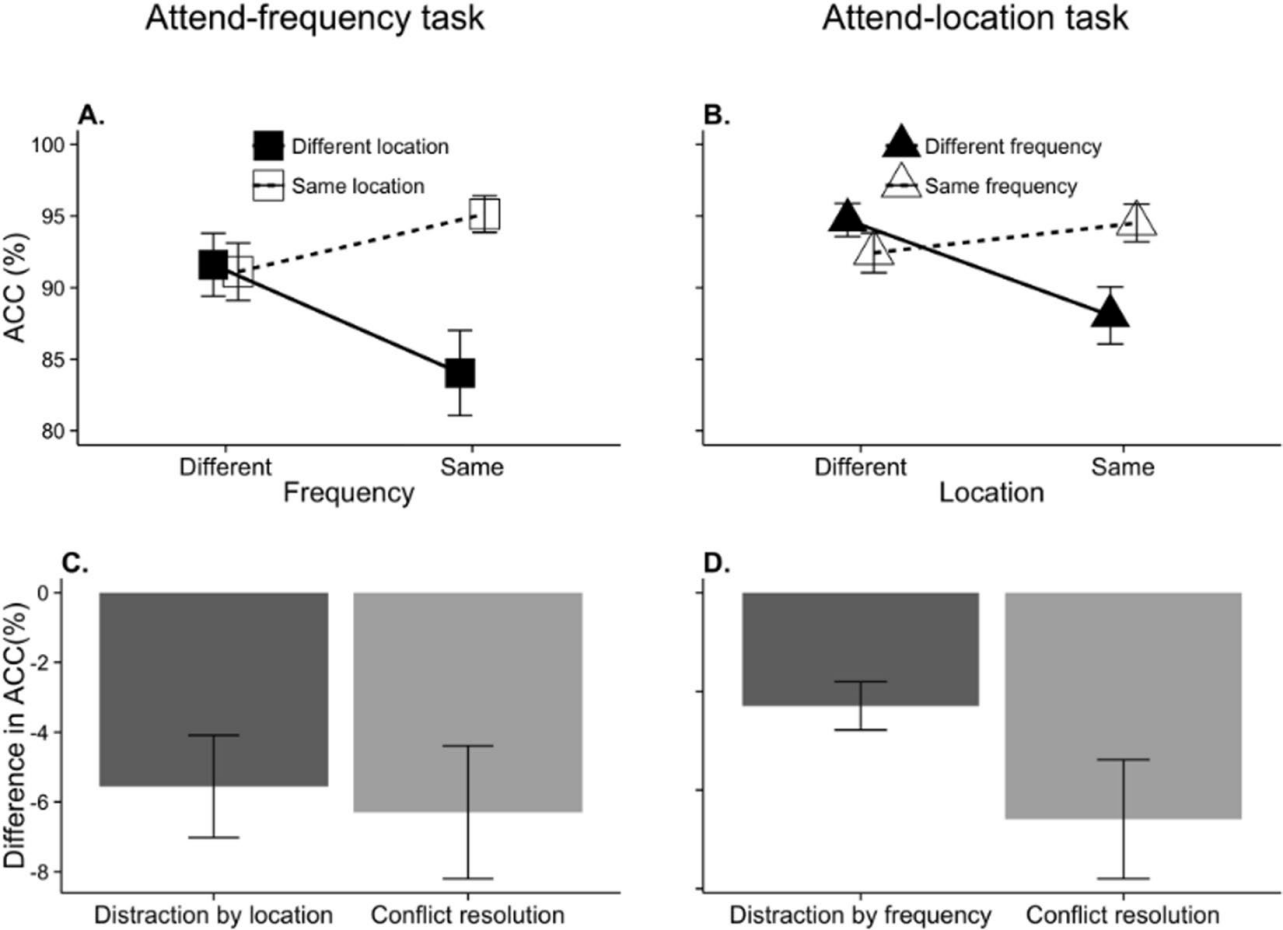
Group mean accuracy for the (**A**) attend-frequency and (**B**) attend-location TAiL tasks. Group mean measures of distraction and conflict resolution for the (**C**) attend-frequency and (**D**) attend-location TAiL tasks. The error bars represent the standard error of the mean.

Similarly for the accuracy data (Fig. 2A,B; Table 2), participants were more accurate when both features remained constant or changes than when only one feature changed (attend frequency: *F*(1, 15) = 8.17, *p* = 0.012, ${{\rm{\eta }}}_{{\rm{p}}}^{2}$ = 0.35; attend location: *F*(1, 15) = 13.02, *p* = 0.003, ${{\rm{\eta }}}_{{\rm{p}}}^{2}$ = 0.47). Post-hoc analysis showed that listeners were more accurate when the task-irrelevant feature changed than when it stayed constant in trials where the task-relevant feature also stayed constant (attend-frequency: *t*(15) = −3.74, *p* = 0.002; attend-location: *t*(15) = −4.08, *p* = 0.001). In the attend-location task it was found that listeners were more accurate when the task-irrelevant feature stayed constant than when it changed in trials where the task-relevant feature changed (*t*(15) = 2.34, *p* = 0.034). This effect was not found in the attend-frequency task (*t*(15) = 0.23, *p* = 0.82).

**Table 2:** Group mean and standard error of the mean accuracy levels from both TAiL tasks for different and same irrelevant feature changes, and incongruent and congruent feature changes.

	Attend-frequency task ACC (%)		Attend-location task ACC (%)	
	Mean	SEM	Mean	SEM
Different irrelevant feature	87.82	1.95	91.39	1.30
Same irrelevant feature	93.13	1.23	93.47	0.96
Incongruent	87.57	1.88	90.24	1.27
Congruent	93.37	1.30	94.62	0.86

#### Distraction

In the attend-frequency task (Fig. 1A; Table 1), RTs were significantly slower for trials where the irrelevant location of the two tones changed than when they stayed constant (*F*(1, 15) = 28.44, *p* < 0.001, ${{\rm{\eta }}}_{{\rm{p}}}^{2}$ = 0.66). Participants were also less accurate (Fig. 2A; Table 2) when the location of the two tones differed (*F*(1, 15) = 12.38, *p* = 0.003, ${{\rm{\eta }}}_{{\rm{p}}}^{2}$ = 0.45). Similarly, in the attend location task, participants were slower (Fig. 1B; Table 1) (*F*(1, 15) = 35.30, *p* < 0.001, ${{\rm{\eta }}}_{{\rm{p}}}^{2}$ = 0.70) and less accurate (Fig. 2B; Table 2) (*F*(1, 15) = 6.35, *p* = 0.024, ${{\rm{\eta }}}_{{\rm{p}}}^{2}$ = 0.30) for trials where the irrelevant-feature of tone frequency changed between the two tones.

In summary, we found behavioral evidence of *conflict resolution* and *distraction* in both the attend-frequency and attend-location tasks (Figs 1C,D and 2C,D).

### Electrophysiological data

**Figure 3:**
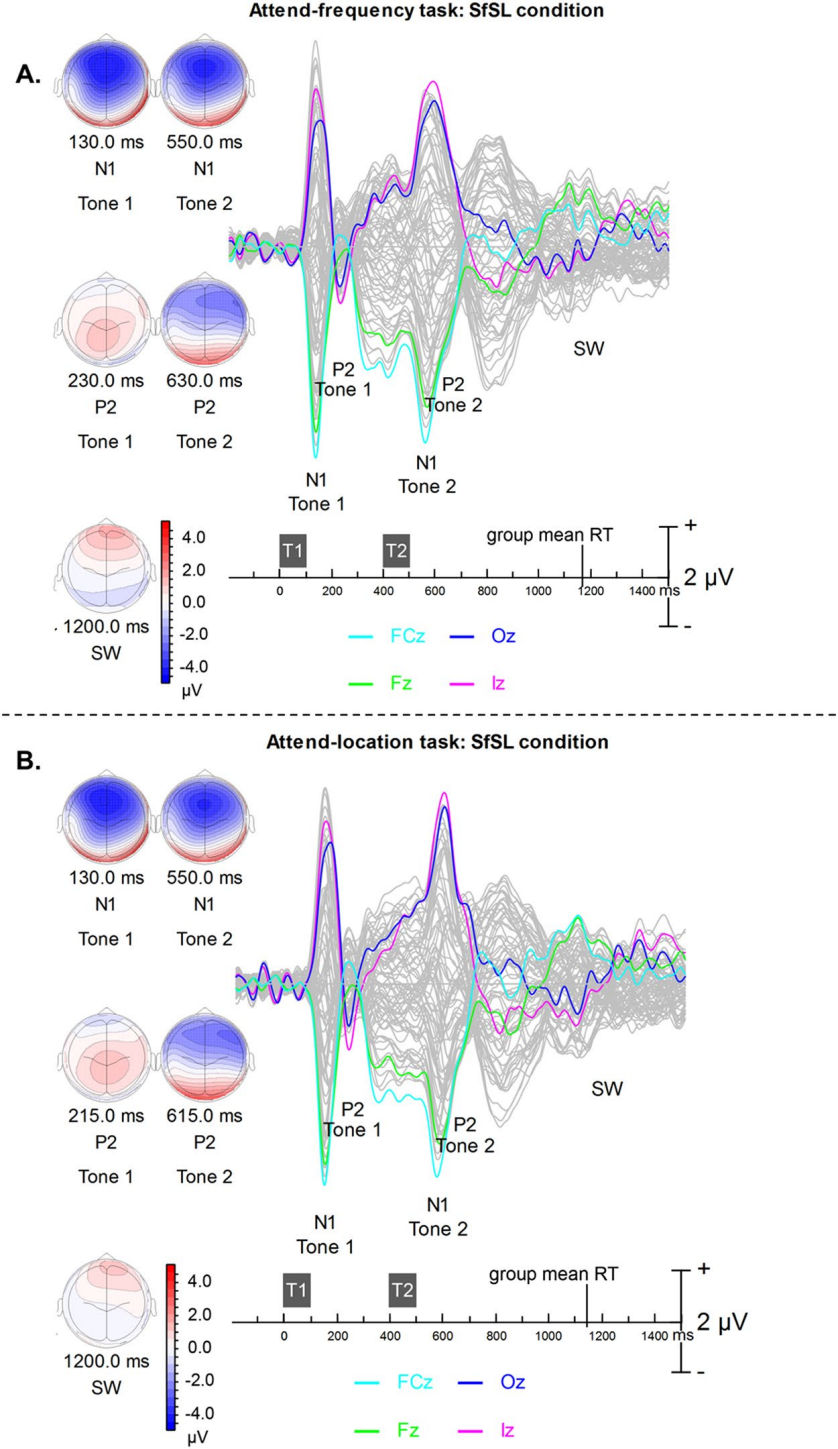
Group mean event-related brain potentials (for 65 channels) averaged over all SfSL trials from (**A**) attend-frequency and (**B**) attend-location TAiL tasks. The FCz electrode is shown in turquoise, Fz in green, Oz in purple and Iz in pink, all other channels are shown in gray. T1 = first tone; T2 = second tone. Isopotential contour maps are shown for the N1 (115 ms and 100 ms, respectively), P2 (200 ms and 200 ms, respectively) for each tone and slow wave (SW). Group mean RT for Same frequency Same location trials was 772 ms for the attend-frequency task and 741 ms for the attend-location task, time-locked to the onset of the second tone.

Figure 3 shows butterfly plots for the *baseline* measure from the attend-frequency and attend-location tasks. In both tasks, the tone pair generated N1 and P2 waves that were time-locked on sound onset. The iso-contour maps show that the N1 wave has a frontocentral scalp distribution and inverted in polarity at mastoid sites, consistent with generators in the superior temporal gyrus along the Sylvian fissure. The P2 wave has a more centro-parietal distribution whereas the slow wave showed a more frontal distribution, with a peak anterior to that of the N1 wave. The auditory evoked responses elicited by the first tone of the pair were little affected by task instructions. The effect of task began after the second tone was presented. All future ERP timings are with reference to the onset of tone 2 (T2) at 400 ms.

#### Conflict resolution

**Figure 4:**
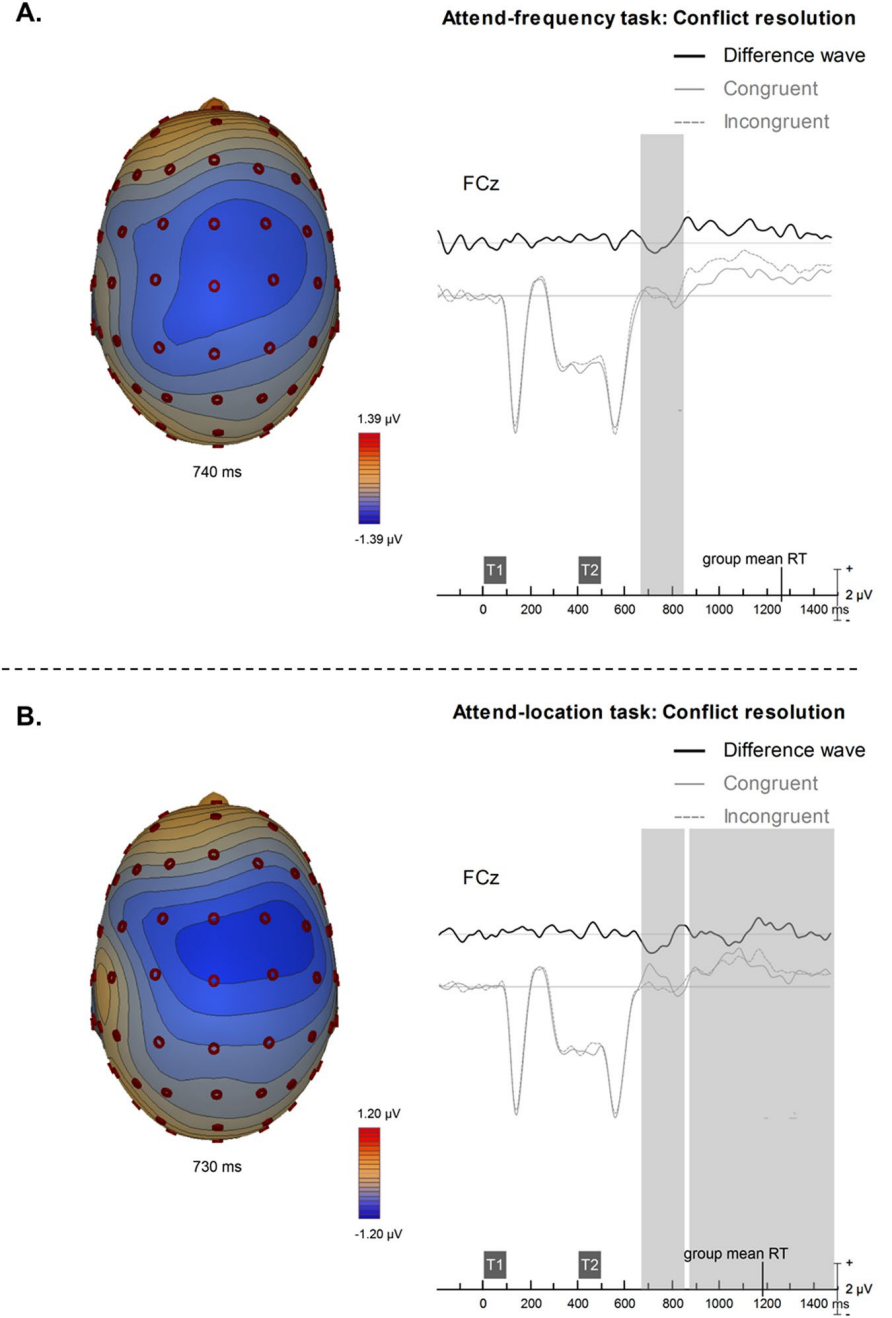
Conflict resolution TAiL effect group mean event-related brain potentials recorded over the central frontal (FCz) scalp region for the (**A**) attend-frequency task and (**B**) attend-location task. T1 = first tone; T2 = second tone. Contour maps illustrate the brain activity at the peak of the difference waves. Shaded areas indicate the time period where the spatio-temporal cluster was significant, p < 0.05. For the attend-frequency task, the spatio-temporal cluster included the following electrodes: FCz, FC2, FC4, Cz, C2, C4, C6, CP2, CP4, CP6, Pz, P2, P4, and P6. For the attend-location, the spatio-temporal cluster included electrodes F4, FC1, FCz, FC2, FC4, FC6, C1, C2, C4, C6, CP2, CP4, CP6, and P4. Group mean RT across trials was 859 ms for the attend-frequency task and 793 ms for the attend-location task, time-locked to the onset of the second tone.

Figure 4 shows the conflict resolution, which is highlighted by contrasting ERPs for congruent trials (where both tone features stay constant or change) with those elicited by incongruent trials (where just one of the tone features change). In the attend frequency task, there was a significant difference, with incongruent trials generating more negative ERPs between 270 and 450 ms after T2 at frontocentral sites (Fig. 4A). In the attend-location task, the incongruent trials generated significant increased in negativity between 270 and 450 ms and again between 475 and 1100 ms over the right frontocentral sites (Fig. 4B).

Post-hoc analysis of this *conflict resolution* effect showed that trials where the task-irrelevant feature changed generated more positive ERPs than when it stayed constant. This effect was found when the task-relevant feature stayed constant in the attend-frequency until 995 ms after T2, peaking at 250 ms, and in the attend-location task until 1025 ms after T2, peaking at 260 ms (Supplementary Material Fig. 1A,B). The effect was also found when the task-relevant feature changed within the trial: between 170–300 ms (peaking at 250 ms) and 535–930 ms after T2 in the attend-frequency task, and between 190 and 680 ms (peaking at 280 ms) after T2 in the attend-location task (Supplementary Material Fig. 2A,B).

We compared the source activity from the frequency and location tasks using Brain Electrical Source Analysis (BESA) software (version 5.3). The analysis was conducted on the *conflict resolution* difference waves. For each participant and condition, we began with a regional source in the left and right temporal lobes near Heschl’s gyrus. Then we optimize the location of both sources simultaneously. The ANOVA on source coordinates was not significant. This was expected given that the congruent trials included a mixture of pitch and location feature changes.

#### Distraction

**Figure 5:**
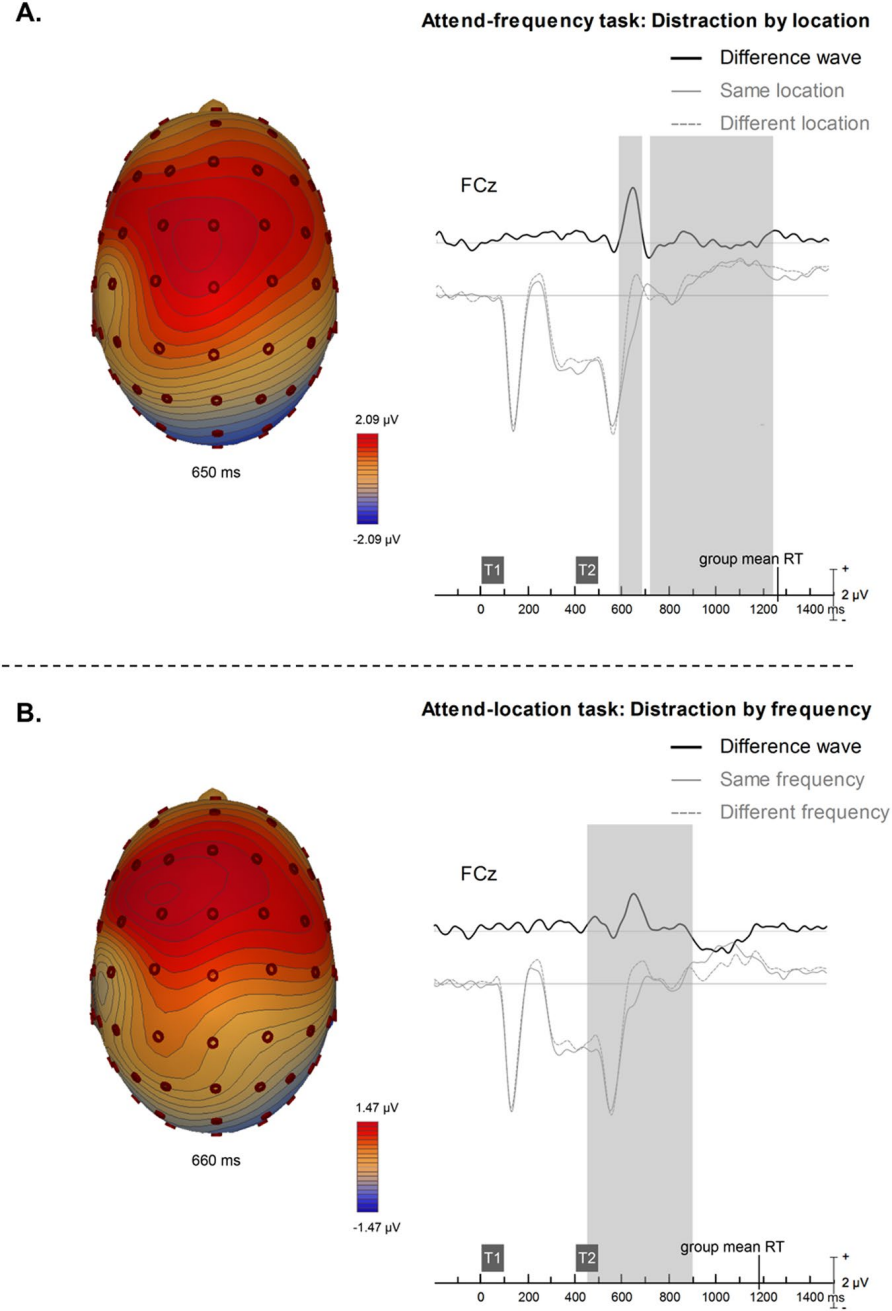
Distraction TAiL effect group mean event-related brain potentials recorded over the central frontal (FCz) scalp region for the (**A**) attend-frequency task and (**B**) attend-location task. T1 = first tone; T2 = second tone. Contour maps illustrate the brain activity at the peak of the difference waves. Shaded areas indicate the time period where the spatio-temporal cluster was significant, p < 0.05. For the attend-frequency task, the spatio-temporal cluster included the following electrodes: AF3, AFz, F5, F3, F1, Fz, F2, F4, F6, FC5, FC3, FC1, FCz, FC2, FC4, FC6, T7, C1, Cz, C2, C4, TP7, CP3, CP1, CPz, CP6, P9, P7, P4, P6, P8, P10, PO7, POz, PO4, PO9, O1, Oz, O2, and Iz. For the attend-location, the spatio-temporal cluster included the electrodes F1, Fz, F2, F4, FC5, FC3, FC1, FCz, FC2, FC4, FC6, C1, Cz, C2, C4, C6, CP1, CP2. Group mean RT across trials was 859 ms for the attend-frequency task and 793 ms for the attend-location task, time-locked to the onset of the second tone.

**Figure 6:**
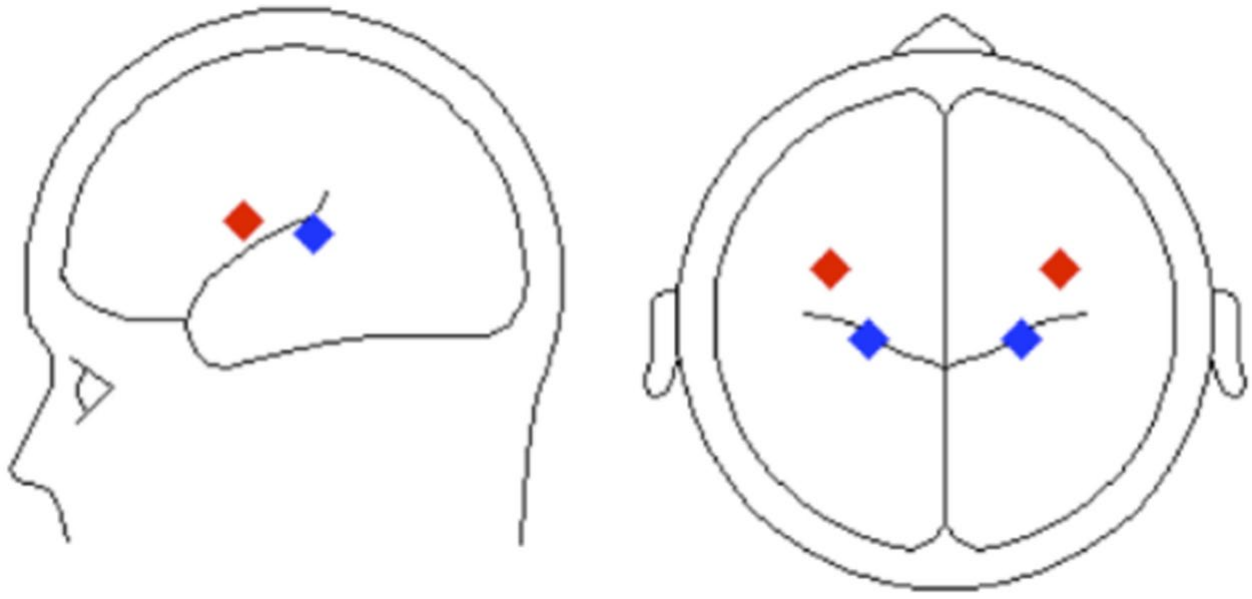
Dipole solution for distraction by location in the attend-frequency task (blue) (mean ± sem: x = 22.9 ± 1.9; y = −25.3 ± 5.4; and z = 13.5 ± 3.0) and distraction by frequency in the attend-location task (red) (mean ± sem: x = 34.7 ± 1.9, y = 1.3 ± 3.9; z = 20.1 ± 1.9).

Figure 5 shows the ERPs elicited when the task-irrelevant feature changed versus when it stayed constant. In both tasks, we found a significant increase in positivity at frontocentral sites (*p* < 0.001; Fig. 5). The polarity of this effect inverted at posterior sites (not shown). In both tasks, the difference wave shows an ERP ranging from around 200–300 ms after the onset of the second tone (T2), peaking at about 250 ms, with the attend-location task ever so slightly later than the attend-frequency task.  Evidence from prior research suggests that processing sound identity and sound location engage to a greater extent the ventral and dorsal pathways, respectively^26^. As for *conflict resolution* we compared the source activity from the frequency and location tasks using discrete source modeling. The analysis was conducted on the *distraction* difference waves. The group mean coordinates (Fig. 6) for each experimental condition were then compared using t-test. At a group level the source location for *distraction* by location (in the attend-frequency task) (mean ± sem: x = 33.60 ± 5.08; y = −9.93 ± 7.34; and z = 44.66 ± 4.77) was more medial and posterior than the source location for *distraction* by frequency (in the attend-location task) (mean ± sem: x = 3.86 ± 9.67; y = 12.84 ± 7.10; z = 47.63 ± 6.43). Paired t-tests on the *x-*, and *y-* were significantly different between the frequency and location tasks, but not for the *z-*axes (*x: t*(14) = −2.27, *p* = 0.040; *y*: *t*(14) = −2.96, *p* = 0.010; *z*: *t*(14) = 0.58, *p* = 0.57).

## Discussion

In both the attend-frequency and attend-location tasks interference effects from the task-irrelevant tone feature were found on RT and accuracy. The EEG data showed that this main effect of *distraction* was qualified by the *conflict* resolution interaction. A change in the task-irrelevant feature significantly *distracted* the listeners regardless of whether the task-relevant feature changed or not within the trial. These behavioral effects coincided with a positive displacement that was best illustrated in the difference wave between ERP elicited by trials when the task-irrelevant tone feature changed and those obtained during trials when the task-irrelevant tone feature did not change. This ERP modulation was maximal at frontocentral sites, and peaked at about 250 ms after the onset of the second tone in TAiL tasks. It shows similarity with the Pd and PTc component observed in visual selective attention tasks^14–18^, but with an earlier latency. The observed ERP modulation also shows similarity with the RP component elicited by to-be-ignored sound information in auditory selective attention tasks^6, 7, 19^. In the present study, the ERP modulation elicited by the distracter tone feature is thought to index the suppression, or even the rejection, of irrelevant stimulus feature when it is found not to match the attentional trace of the task-relevant stimuli.

In both visual and auditory tasks these *distraction* components have been shown to present in the area that would be enhanced if the distracter were the target^18^. For example, Degerman *et al*.^6^ found that during a continuous stream of auditory tones, when the listener was told to attend to sounds with a specific relevant feature (e.g. in the left or right ear or with high or low pitch), the RP component’s source activity varied as a function of the irrelevant sound feature. This result was mirrored in the present study, with significantly different regional sources of the *distraction* component reflecting the task-irrelevant feature. That is, regional sources indexing the processing of the task-irrelevant location feature was more posterior in the attend-frequency task than the ones used to account for the task-irrelevant frequency feature effect in the attend-location task. The regional sources for the attend-frequency’s task-irrelevant location feature were also more medial and inferior than the ones associated with the processing of the task-irrelevant frequency feature during the attend-location condition. These differences were unexpected and further research is needed to replicate these effects. The anterior-posterior difference in source location as a function of sound identity (pitch) and sound location is consistent with the ‘what’ and ‘where’ pathway model of auditory scene analysis^26, 30–32^, with the irrelevant location stimuli feature activating a more posterior network in the attend-frequency task than the irrelevant frequency feature in the attend-location task. In the present study, we purposely used regional sources to test the hypothesis that processing sound identity and sound location recruit different auditory areas along the superior temporal gyrus. Such an approach does not depend on identifying the exact source locations, but rather utilizes discrete source analysis as a mean to test current model of auditory processing. Further research using functional magnetic resonance imaging is needed to identify the brain regions or network of brain regions suppressing the distractor stimulus feature.

The RP *distraction* component, in this and other auditory selective attention studies, occurs within an earlier time frame than comparable visual selective attention studies^6, 11^. An earlier effect with auditory stimuli has also been shown in the Stroop task. Visual Stroop tasks typically report an onset of 350–400 ms with a peak at 450 ms post-onset of the stimuli^20–23^. Comparably, auditory Stroop tasks show an earlier onset of 200–250 ms with a peak at around 300 ms^11, 12^.

In this study the *conflict resolution* component was found to occur between 270 and 450 ms in both TAiL tasks. It may be that the auditory stimuli used in TAiL causes earlier component onset than comparable studies in the visual modality as the time from stimulus onset to processing in the primary sensory cortex is markedly shorter in the auditory system when compared to the visual system^33^.

However, a further negative frontocentral component was found to occur 475–1110 ms post second tone onset in the attend-location task’s *conflict resolution* measure. Whilst the distribution of the *conflict resolution* component in the attend-frequency task is similar to that of previous auditory Stroop studies^11, 12^, the timing of this component in the attend-location task straddles the time frames reported in both the auditory and the visual modalities. Therefore these results provide continuing support to the behavioural results found by Stewart and Amitay^25^ whereby TAiL’s attend-frequency *conflict resolution* measure focuses on auditory-specific non-spatial attention, whilst the attend-location *conflict resolution* measure assesses supramodal spatial based attention.

The design of TAiL is advantageous firstly because the stimuli used in the two TAiL tasks (attend-frequency and attend-location) are the same, just the instructions change, therefore reflecting purely cognitive processing differences rather than contrasting stimuli. Secondly, the underlying brain activation of the *distraction* and *conflict resolution* measures are calculated from the same blocks of trials allowing them to be directly compared. The results discussed in this paper suggest that through TAiL’s simple paradigm, it is possible to assess a listener’s ability to deal with auditory-specific and supramodal sensory *conflict resolution* between relevant and irrelevant stimulus features. Furthermore, results indicate that the *distraction* measure does indeed result from the irrelevant, to be ignored stimuli, rather than the stimulus feature the listener is basing their decision upon.

The present study aimed to evaluate listeners’ ability to suppress irrelevant sound information and to resolve conflict concurrently, as is often required in everyday listening environments. The results indicate that the TAiL paradigm succeeds in assessing both of these skills simultaneously and display distinct neural correlates. In addition, by simply changing the task goal, via a change in instructions, the timings and distributions of the *distraction* and *conflict resolution* measures shift. The TAiL paradigm therefore has potential use in clinical populations (e.g. children with Auditory Processing Disorder or older listeners with hearing difficulties) as a simple test to separate cognitive listening ability from sensory ability.

## Method

### Participants

Sixteen participants aged 18–25 (Mean = 22.25 years, SD = 2.26 years; 7 females and 9 males) were recruited through the Rotman Research Institute participant database. Inclusion criteria was normal hearing (thresholds below 25 decibel (dB) hearing level (HL) bilaterally at frequencies between 250 and 8000 Hz, inclusive, and a normal score (0–3) on the Quick Speech-in-Noise test (QuickSIN^34^). Exclusion criteria was any self-reported history of brain damage, brain surgery, history of language-related or attention-related conditions, autism spectrum disorders or any auditory system disorders (e.g. Auditory Processing Disorder). Informed consent was signed by each participant prior to the experiment and each was paid at a rate of $15 per hour. All methods were approved by, and performed in accordance with the relevant guidelines and regulations of, Toronto Academic Health Services Network.

### Stimuli and Task

All tones were made up of sinusoids with a duration of 100 ms, gated on/off by 10 ms cos ramps, and were presented monaurally at 65 dB sound pressure level (SPL) using Etymotic ER-3A insert-earphones (Etymotic Research, Elk Grove, IL, USA). Tone frequency was randomly selected from the range 476.18–6187.50 Hz with at least 2.1 equivalent rectangular bandwidths (ERBs; ~4 semitones) between the trial’s tones, therefore making it well within the listener’s ability to discriminate between different frequencies^35^. The inter-trial-interval varied randomly between 500 and 1000 ms, whilst the inter-stimulus interval (ISI) was set at 300 ms.

Listeners were asked to respond as fast and as accurate as possible after the second tone. Responses less than 200 ms and more than 2500 ms were excluded from further analysis in case of premature responding and lapses of attention or interruption of performance. As soon as the listener responded to each trial (attend-frequency task group mean: 859 ms; attend-location task group mean 793 ms) visual feedback was provided for 500 ms. If they answered correctly a smiley face was displayed. If they answered incorrectly the same face was shown with a sad expression.

TAiL stimuli were automated and presented in Matlab 2012b using PsychoPhysics Toolbox v3.0.10, a SoundMAX integrated digital HD Audio sound card. All behavioural responses were made using two buttons on the keyboard (the letters ‘Q’ and ‘E’) with the hand of the participant’s choice. Listeners were tested individually in a sound-attenuated booth.

Regardless of the task type in TAiL, the stimuli and paradigm remained the same, just the instructions to the participant changed. In each trial, participants heard a tone pair where the individual tones were either the same or different in frequency and/or spatial location (ear presentation) (e.g. SfSL - same frequency and same location; DfSL - different frequency and same location; etc.). In both attend-frequency and attend-location the listener had to indicate via a button press if the task-relevant sound feature (i.e. the location of the two tones in the attend-location task) were same or different, whilst ignoring the task-irrelevant sound feature (i.e. the frequency of the two tones in the attend-location task). A full description of the task and stimuli can be found in Zhang *et al*.^13^.

From the attend-frequency and attend-location tasks measures of *baseline*, *conflict resolution* (the ability to deal with conflicting sound feature information) and *distraction* (involuntary orientation to the irrelevant sound feature) are calculated. The *baseline* measure is calculated for each task type from trials where both sound features, frequency and location, remain constant (i.e. SfSL trials). The *conflict resolution* is calculated as the difference between trials where the task-relevant and task-irrelevant features are incongruent and congruent (i.e. trials where the frequency and the location are both same/different minus trials where one feature is the same and the other different). The *distraction* measure is calculated as the difference in accuracy and response time between conditions where the task-irrelevant feature was either constant or changed between Tone 1 and Tone 2 (e.g. in the attend-location task – behavioral responses to trials where the frequency of the two sounds stayed the same was subtracted from trials in which the frequency changed).

### Procedure

Both tasks (attend-frequency and attend-location) were repeated nine times. Each block included 40 trials, providing a total of 360 trials per task condition per participant. The order of the task types was alternated across the blocks, and counterbalanced across participants. For the first block of the attend-frequency and attend-location tasks there was a practice block prior. Each practice block involved five trials, accompanied by on-screen instructions. Participants had to reach 60% accuracy or more to move onto the full testing block. Instructions to define the task type and to ask the participant to be as fast and as accurate as possible were provided at the start of each block and visual feedback regarding the listener’s performance was provided after each trial throughout. The total testing/recording time lasted ~45 minutes.

### Behavioural analysis

RTs (ms) from correct trials, and accuracy (% correct) were used in the analysis. Repeated measure ANOVAs with the task-relevant and task-irrelevant dimensions as repeated measures were run for each TAiL task. Conflict resolution was the interaction between the relevant and irrelevant measures (i.e. comparing incongruent feature trials to congruent feature trials), and distraction was the main effect of the task-irrelevant measure (i.e. comparing different task-irrelevant feature trials to same task-irrelevant feature trials).

### EEG recording and analysis

EEG was recorded from 66 scalp electrodes using a BioSemi Active Two acquisition system (BioSemi V.O.F., Amsterdam, The Netherlands). The electrode montage was according to the BioSemi electrode cap based on the 10/20 system and included a common mode sense active electrode and driven right leg passive electrode serving as ground. Ten additional electrodes placed below the hairline (both mastoid, both pre-auricular points, outer canthus of each eye, inferior orbit of each eye, two facial electrodes) to monitor eye movements and to cover the whole scalp evenly. The latter is important because we used an average reference (i.e. the average of all scalp EEG channels as the reference for each EEG channel) for ERP analyses. Neuroelectric activity was digitized continuously at rate of 512 Hz with a bandpass of DC-100 Hz, and stored for offline analysis. All off-line analyses were performed using BESA (version 5.3; MEGIS GmbH, Gräfelfing, Germany).

The continuous EEG data were first digitally filtered with 0.1 Hz high-pass (forward, 6 dB/octave) and 20 Hz low-pass filters (zero phase, 24 dB/octave) using BESA software. The analysis epoch consisted of 200 ms of pre-stimulus activity and 1500 ms of post-stimulus activity time-locked to the onset of the first tone, from trials where the participant answered correctly. For each participant, a set of ocular movements was obtained prior to and after the experiment^36^. From this set, averaged eye movements were calculated for both lateral and vertical eye movements as well as for eye-blinks. A principal component analysis of these averaged recordings provided a set of components that best explained the eye movements. The scalp projections of these components were then corrected from the experimental ERPs in order to minimize ocular contamination, using BESA software (version 5.3). Epochs contaminated by excessive deflections (greater than ± 120 uV anywhere in the epoch) after correcting for ocular contaminations were excluded from the averages. For each participant, the remaining epochs were averaged according to electrode position and experimental condition (i.e., SfSL, SfDL, DfSL, DfDL). Each average was baseline-corrected with respect to the pre-stimulus interval. The group mean proportion of trials included in the ERP average for each TAiL condition was 84.27% (SD = 11.06%) in the attend-frequency task and 85.15% (SD = 8.81%) in the attend-location task. One male participant was excluded due to a high number of artifacts during recording.

BESA statistics 2.0 was used as a data-driven method to compare two conditions over all scalp regions up to 1500 ms post onset of the first tone. A two-stage analysis first computed a series of t-tests that compared the ERP amplitude between the conditions at every time point from 0 to 1500 ms after the onset of Tone 1. This preliminary step identified clusters both in time (adjacent time points) and space (adjacent electrodes) where the ERPs differed between the conditions. The channel diameter was set at 4 cm which led to around 4.03 neighbours per channel. We used a cluster alpha of 0.05 for cluster building. In the second stage of this analysis, all p-values reported for each data point were corrected for multiple comparisons using a Monte-Carlo resampling technique^37^. The number of permutations was set at 1,000 with a 0.05 threshold.

We also performed regional source analyses using BESA software (version 5.3) on the *distraction* difference waves and compared source locations for the frequency and location tasks using student t-test. The source location was optimized for a 60 ms interval centered on the peak of the difference wave for each task. For each participant and condition, we began with a regional source in the left and right temporal lobes near Heschl’s gyrus. During optimization, the location of the left and right regional source was forced to be symmetric. We used a four shell head model with head radius of 85 mm, and thickness for scalp, bone and cerebrospinal fluid of 6, 7, 1 mm, respectively. The relative conductivities were 0.33, 0.33, 0.0042, and 1 for brain, scalp, bone and cerebrospinal fluid, respectively.

## Electronic supplementary material

Supplementary material
