# Supplementary material for: Neural correlates of distraction and conflict resolution for nonverbal auditory events
Source: Sci Rep. 2017 May 9;7:1595. doi: 10.1038/s41598-017-00811-7 (PMC5431653; doi:10.1038/s41598-017-00811-7)
Supplement: Supplementary file 1 — Supplementary material [file 41598_2017_811_MOESM1_ESM.doc]

Supplementary material

***Neural correlates of distraction and conflict resolution for nonverbal auditory events***

Hannah J Stewart, Sygal Amitay, and Claude Alain

**Conflict resolution post hoc analysis**

**Figure 1**: Conflict resolution TAiL effect post-hoc group mean event-related brain potentials when the task-relevant feature remained constant during the trial. Recorded over the central frontal (FCz) scalp region for the (A) attend-frequency task and (B) attend-location task. T1 = first tone; T2 = second tone. Contour maps illustrate the brain activity at the peak of the difference waves. Shaded areas indicate the time period where the spatio-temporal cluster was significant, p < .05. For the attend-frequency task, the spatio-temporal cluster included the following electrodes: F5, F3, F 1, F2, F4, F6, FC5, FC3, FC1, FCz, FC2, FC4, FC6, C1, Cz, C2, C4, C6, CP3, CP1, CPz, CP2, CP4, PO4, P3, P1, Pz, P2, P4 and T7. For the attend-location, the spatio-temporal cluster included the electrodes AF3, AFz, AF4, F5, F4, F6, F8, FT7, FT8, FC5, FC3, FC4, FC6, C4, C6, T7, T8, CP3, CP1, CPz, CP2, CP4, CP6, Cz, C2, P9, P7, P5, P3, P1, Pz, P4, PO7, PO3, POz, O1, Oz and O2. Group mean RT across trials was 859 ms for the attend-frequency task and 793 ms for the attend-location task, time-locked to the onset of the second tone.

**Figure 2**: Conflict resolution TAiL effect post-hoc group mean event-related brain potentials when the task-relevant feature changed during the trial. Recorded over the central frontal (FCz) scalp region for the (A) attend-frequency task and (B) attend-location task. T1 = first tone; T2 = second tone. Contour maps illustrate the brain activity at the peak of the difference waves. Shaded areas indicate the time period where the spatio-temporal cluster was significant, p < .05. For the attend-frequency task, the first spatio-temporal cluster included the following electrodes: FC4, C1, C2 and C4; and the second spatio-temporal cluster: F1, Fz, F2, F4, F6, FC1, FCz, FC2, FC4, C1, Cz, C2, C4, CP1 and CPz. For the attend-location, the first spatio-temporal cluster included the electrodes F1, Fz, F2, F4, F10, FC5, FC3, FC1, FCz, FC2, FC4, FC6, C1, C4 and C6; and the second spatio-temporal cluster: C1, Cz, C2, CP3, CP1 and CPz. Group mean RT across trials was 859 ms for the attend-frequency task and 793 ms for the attend-location task, time-locked to the onset of the second tone.


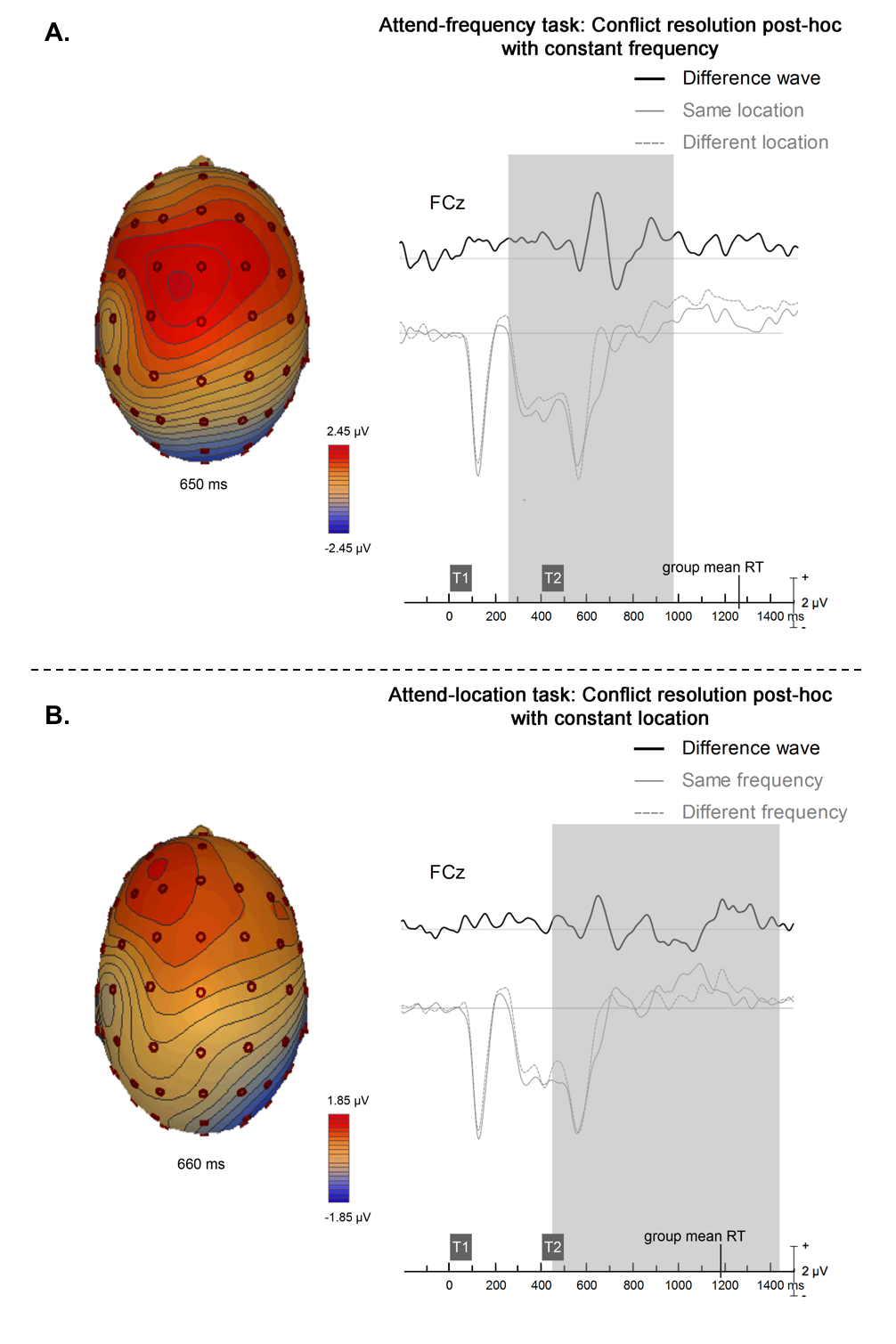


**Figure 1**


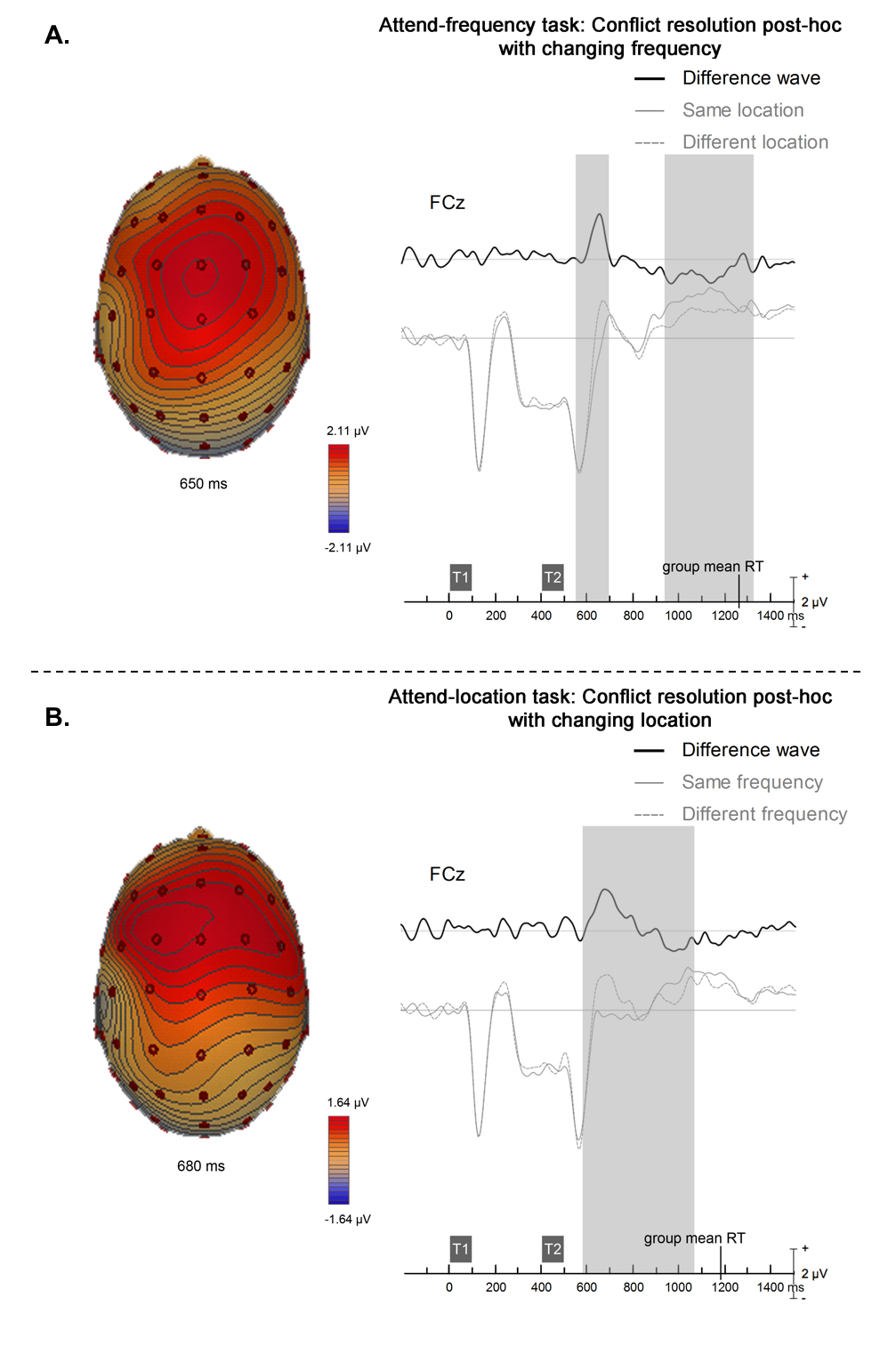


**Figure 2**
